# Compartment Syndrome as a Result of Systemic Capillary Leak Syndrome

**DOI:** 10.1155/2016/4206397

**Published:** 2016-09-05

**Authors:** Kwadwo Kyeremanteng, Gianni D'Egidio, Cynthia Wan, Alan Baxter, Hans Rosenberg

**Affiliations:** ^1^The Ottawa Hospital, Ottawa, ON, Canada; ^2^Division of Critical Care, Department of Medicine, The University of Ottawa, Ottawa, ON, Canada; ^3^School of Psychology, The University of Ottawa, Ottawa, ON, Canada; ^4^Department of Anesthesiology, The University of Ottawa, Ottawa, ON, Canada; ^5^Department of Emergency Medicine, The University of Ottawa, Ottawa, ON, Canada

## Abstract

*Objective*. To describe a single case of Systemic Capillary Leak Syndrome (SCLS) with a rare complication of compartment syndrome.* Patient*. Our patient is a 57-year-old male, referred to our hospital due to polycythemia (hemoglobin (Hgb) of 220 g/L), hypotension, acute renal failure, and bilateral calf pain.* Measurements and Main Results*. The patient required bilateral forearm, thigh, and calf fasciotomies during his ICU stay and continuous renal replacement therapy was instituted following onset of acute renal failure and oliguria. Ongoing hemodynamic (Norepinephrine and Milrinone infusion) and respiratory (ventilator) support in the ICU was provided until resolution of intravascular fluid extravasation.* Conclusions*. SCLS is an extremely rare disorder characterized by unexplained episodic capillary hyperpermeability, which causes shift of volume and protein from the intravascular space to the interstitial space. Patients present with significant hypotension, hemoconcentration, hypovolemia, and oliguria. Severe edema results from leakage of fluid and proteins into tissue. The most important part of treatment is maintaining stable hemodynamics, ruling out other causes of shock and diligent monitoring for complications. Awareness of the clinical syndrome with the rare complication of compartment syndrome may help guide investigations and diagnoses of these critically ill patients.

## 1. Introduction

Systemic Capillary Leak Syndrome (SCLS, Clarkson Syndrome) was first described by Clarkson et al. in 1960 [1]. SCLS is an extremely rare disorder characterized by unexplained episodic capillary hyperpermeability, which causes shift of volume and protein from the intravascular space to the interstitial space. Patients present with significant hypotension, hemoconcentration, hypovolemia, and oliguria. Severe edema results from leakage of fluid and proteins into tissue. The etiology of SCLS is not known. The most frequent association between patients is an IgG monoclonal gammopathy. There have been approximately 100 reported cases since 2007. Most patients were previously healthy and middle-aged, and both sexes appear equally represented [2–4]. A familial component has not been identified [5].

## 2. Case Report

Our patient is a 57-year-old male, referred to our hospital from a neighboring community hospital due to polycythemia (hemoglobin (Hgb) of 220 g/L), hypotension, and bilateral calf pain. His past medical history was significant for gastroesophageal reflux disease and hypertension. In the 5 weeks prior to presentation, he had begun an exercise program, which included running on a treadmill, and over the last week he complained of progressive bilateral leg swelling and pain. Additionally, he experienced significant nausea and weakness over that same time. In the two days prior to presentation, the bilateral calf pain and swelling rapidly progressed resulting in the “worst pain of his life.” The remaining review of systems was unremarkable.

On presentation to our Emergency Department, the patient was alert and oriented, his vital signs were initially stable including blood pressure of 150/99 (blood pressure in community hospital noted to be 68/40 before receiving 2 L bolus of normal saline), and his physical exam was unremarkable except for bilateral swollen calves, described as “rock hard” and tender to palpation. There was also limited range of motion at his ankle, with severe pain on plantar and dorsiflexion. At this point, compartment syndrome was suspected and the Orthopedics team was consulted. This diagnosis was confirmed by compartment pressure measurements of 110 mmHg and the patient was booked for emergent bilateral fasciotomies.

His initial blood work showed Hgb of 210 g/L, WBC of 27.3 × 10^9^/L, albumin of 26 g/L, and creatinine of 135 umol/L (see Table 1). Due to his elevated Hgb, phlebotomy of 600 cc was performed prior to his operation. Throughout the operation, the patient's blood pressure was unstable despite significant crystalloid fluids administered (3 L of normal saline) requiring institution of vasopressor support. Following his bilateral fasciotomies, the patient remained hypotensive and difficult to wean from the ventilator, resulting in his admission to the intensive care unit.

**Figure 1:**
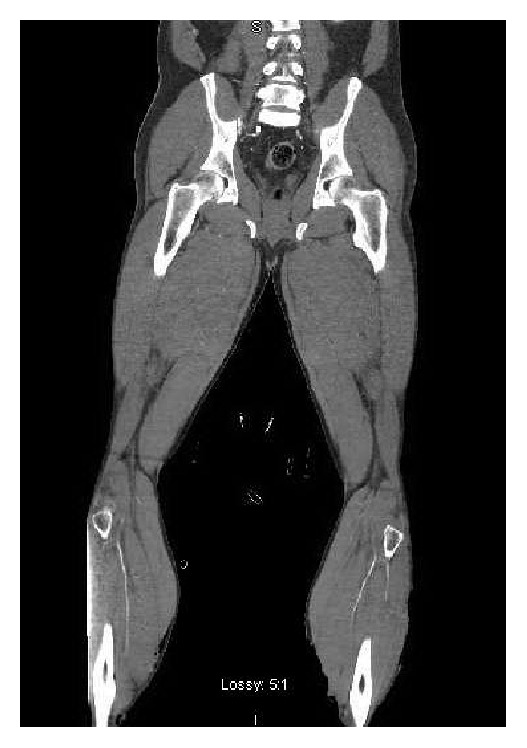
CT angiogram of the lower limbs showing marked narrowing of the distal vessels (arterial and venous) but no occlusive pathology.

His initial resuscitation included administration of 6 liters of crystalloid over the first 12 hours with further boluses of albumin 5% for a total of 2.5 L, continued vasopressor support (Norepinephrine titrated to MAP > 65), broad spectrum antibiotic coverage (Pip-Tazo 3.375 g q6 h), and parenteral steroids (Solucortef 100 mg IV q8 hr) while working up the cause of his ongoing shock. Measures of our patient's Cardiac Index (CI) using the CardioQ*™* esophageal device showed significant decline in cardiac function with CIs of 0.9–1.6 L/min during his initial presentation, prompting initiation of a Milrinone infusion. An urgent CT angiogram of the lower limbs was performed which showed marked narrowing of the distal vessels (arterial and venous) but no occlusive pathology (Figure 1).

Due to oliguria, increasing creatinine, CK (initially 1353 U/L and rising to 11590 U/L by postoperative day one), and positive urine myoglobin, the patient was thought to be in acute renal failure secondary to relative intravascular hypovolemia and rhabdomyolysis. For this reason, continuous renal replacement therapy was started and continued for three days.

Continuing intravascular fluid extravasation on postoperative days one and two necessitated performance of bilateral fasciotomies of the thighs and forearms. Continued ventilator support was required for eleven days related to his gross edema. He had mild pulmonary edema, moderate bilateral effusions, and mild ascites. On further work up, the patient was found to be JAK-2 mutation negative, with normal C3 and C4 complement levels, and serum immunofixation showed a monoclonal IgG band.

Given the initial presentation of hemoconcentration, hypoalbuminemia, and hypotension, with the concomitant presence of the monoclonal IgG band, a diagnosis of Systemic Capillary Leak Syndrome (Clarkson Syndrome) was made. Our patient eventually stabilized, allowing for admission to the Orthopedic service, followed by a discharge home with minimal sequelae other than a persisting bilateral median and ulnar nerve neuropathies. Hemoglobin and albumin were 93 g/L and 23 g/L, respectively.

## 3. Discussion

### 3.1. Clinical Features

SCLS typically presents in three phases: a prodromal, an extravasation, and a recovery phase [4, 5]. In prodromal phase, the patient presents with signs typical of a viral illness such as lethargy, fever, nausea, and vomiting. During the extravasation phase, the patient shows signs of increased capillary permeability, such as significant generalized edema and hypotension. The edema can be profound, manifesting as pleural effusions, pericardial effusions, and rarely cerebral, epiglottic, and macular edema [2]. In the most severe cases, tissue edema can lead to compartment syndrome requiring fasciotomies; however, this is a rare complication. Moreover, tissue edema can also lead to rhabdomyolysis, and hypotension may be considerable.

Despite being edematous, the patient may require several liters of fluid and vasoactive agents to maintain adequate blood pressure. Acute renal failure can also occur secondary to hypovolemic shock. The recovery phase of SCLS is associated with the mobilization of extravascular fluid. This point is when the patient is at high risk of developing pulmonary edema.

### 3.2. Lab Features

High hemoglobin and hematocrit are present in almost all cases. Serum protein and albumin are also low. Elevated CK secondary to rhabdomyolysis is also seen. A monoclonal protein is present on serum electrophoresis in most cases. One review stated that there was no monoclonal protein detected in only 10 cases [2].

### 3.3. Pathophysiology

The pathophysiology of SCLS has not been established. Imaging with radio labelled albumin and dye has shown increased capillary permeability which can result in loss of up to 70% of total intravascular volume [5]. Polycythemia takes place because the RBCs are too large to permeate through the capillaries and often the degree of polycythemia reflects the severity of illness. Unfortunately, these episodes can reoccur spontaneously. It has been hypothesized that higher levels of circulating factors associated with capillary permeability such as coagulation factors, bradykinin generation, complement, serotonin, prostaglandins, and histamine levels would be associated with SCLS. These factors do not appear to be altered in patients with SCLS [4, 6]. Some medications (gemcitabine, retinoids, sirolimus, and interferon-*α*) are among the rare drug-induced causes of SCLS.

### 3.4. Differential Diagnosis

It is essential to rule out other forms a shock, that is, distributive (sepsis/anaphylaxis/pancreatitis), cardiogenic, obstructive, and other forms of hypovolemia (bleeding, etc.). Although edemas may not be present in these cases, it is important to rule them out because lack of recognition can be fatal. Other rare conditions such as C1-esterase deficiency and systemic mastocytosis can present like SCLS. As in our case, SCLS can be mistaken as polycythemia vera, possibly resulting in phlebotomies which can worsen the patient's shock. An important differentiating factor is that polycythemia vera is not associated with hypotension.

### 3.5. Treatment

The most important part of treatment is maintaining stable hemodynamics. Due to the severe loss of intravascular volume, aggressive fluid resuscitation is often necessary. There does not appear to be any benefit whether crystalloids or colloids are used [7]. We favoured the use of colloids for volume expansion in the hope that the fluid would remain intravascularly for longer. In our case, there was difficult balance between maintaining adequate volume expansion and increasing edema causing worsening compartment syndrome. Fortunately compartment syndrome requiring fasciotomy is rare. During the recovery phase when the patient begins mobilizing fluid, diuretics may be considered to prevent pulmonary edema (assuming stable renal function). There is no proven therapy to prevent future occurrences. Multiple therapies including theophylline and terbutaline, steroids, IVIG, and plasmapheresis have not shown clear benefit.

In terms of prognosis, the progression to multiple myeloma appears rarely [8]. The average of recurrences is about three [2]. Regrettably, 5-year mortality of SCLS has been as high as 76% [9]; but in the most recent reviews [2, 4], in cases between 1990 and 2006, mortality rates were between 25 and 30%. Differences in mortality rate appear to be strongly impacted by the treatment administered to the patients. In a recent series of case studies, Gousseff et al. [10] reported that five years after diagnosis, survival was 85% in 23 patients who had received prophylactic treatment whereas the survival rate plummeted to 20% for five patients who had not. Increasing awareness of SCLS may account for the decreased mortality rates.

## Figures and Tables

**Table 1:** Laboratory assessments, patient results, and the expected range of analyses.

Laboratory analyses	Results	Normal range
White blood cells (WBC)	27.3 × 10^9^/L	3.0–10.5
Hemoglobin (Hgb)	210 g/L	130–170
Platelets (PLAT)	296 × 10^9^/L	125–400
International normalized ratio (INR)	1.3	0.9–1.2
Partial thromboplastin time (PTT)	47 s	22–33

Sodium (Na)	135 mmol/L	136–144
Potassium (K)	4.7 mmol/L	3.6–5.1
Chloride (Cl)	113 mmol/L	101–111
Carbon dioxide (CO2)	14 mmol/L	22–32
Glucose random	10.3 mmol/L	3.8–11.0
Urea	10 mmol/L	2.9–7.1
Creatinine	135 mmol/L	62–106

Albumin	26 g/L	35–48
Lactate	2.7 mmol/L	0.5–2.2
CK	1353 U/L	20–215

Blood gas		
Source	Arterial	
Blood pH	7.14	
Blood pCO2	36 mm Hg	
Blood pO2	162 mm Hg	
Bicarbonate	12 mmol/L	
O2 saturation (calculated)	99 (% O2SAT)	
O2 saturation (measured)	99 (% O2SAT)	
% oxyhemoglobin (FO2Hb)	97%	
Base excess	−15.8 mmol/L	
